# Pacemaker lead malposition in the left atrial roof is masked by normal pacing thresholds

**DOI:** 10.1186/1749-8090-9-53

**Published:** 2014-03-20

**Authors:** Claus Rath, Martin Andreas, Caesar Khazen, Dominik Wiedemann, Andreas Habertheuer, Alfred Kocher

**Affiliations:** 1 Division of Cardiac Surgery, Vienna General Hospital, Medical University of Vienna, Waehringer Guertel 18-20, Vienna A-1090, Austria

**Keywords:** Pacemaker, Lead malpositioning, Lead perforation, Cardiac tamponade, Chest X-ray

## Abstract

Pacemaker lead malpositioning with subsequent cardiac tamponade is a rare, but serious adverse event. We herein report a case of pacemaker lead malpositioning in a 76-year old female caucasian patient. The lead was malpositioned into the roof of the left atrium after perforation of the superior vena cava, resulting in cardiac tamponade. After fast surgical revision and an uneventful post-operative period, the patient was discharged in excellent condition.

## Background

The number of pacemaker implantations is increasing worldwide. The complication rates range between 1.7 [1] and 12.4% [2]. Lead associated complications include migration, venous thrombosis, infection, dislocation, malposition and perforation.

Our case is not a result of subacute or chronic lead migration, but has to be regarded as a procedural adverse event. Perforation of the superior vena cava resulted in cardiac tamponade.

## Case presentation

A 76-year old female caucasian patient was admitted to our department, a tertiary care centre, two hours after undergoing pacemaker implantation for Sick Sinus Syndrome in a peripheral hospital.

She presented in poor haemodynamic condition with pericardial tamponade, requiring mechanical CPR at the entrance of the operation-theatre. Full median sternotomy was performed due to the acuteness of the situation. Opening of the pericardium resulted in hemodynamic stabilization of the patient. At inspection of the heart, no active source of bleeding was found. After insertion of a mediastinal drainage and wound closure, the patient was transferred to the intensive care unit.

The first post-operative day was uneventful. Lead values including voltage levels of P-wave and R-wave, impedance and threshold in pacemaker control were within the normal range (Table 1).

**Table 1:** **Pacing parameters before revision, after revision and normal ranges**[3]**; mV Millivolt; Ω Ohm; V Volt; ms Milliseconds**

		**Before revision**	**After revision**	**Normal ranges**
Sensing	P-wave	6.4 - 6.9 mV	2.1 mV	≥1.5 mV
	R-wave	9.1 - 14.7 mV	14.6 mV	≥5.0 mV
Impedance (bipolar)	Atrial	760 Ω	461 Ω	400 - 1000 Ω
	Ventricular	526 Ω	861 Ω	400 - 1000 Ω
Threshold	Atrial	0.4 V at 0.4 ms	0.6 V at 0.5 ms	<1 V at 0.5 ms
	Ventricular	0.5 V at 0.4 ms	0.5 V at 0.5 ms	<1 V at 0.5 ms

**Figure 1:**
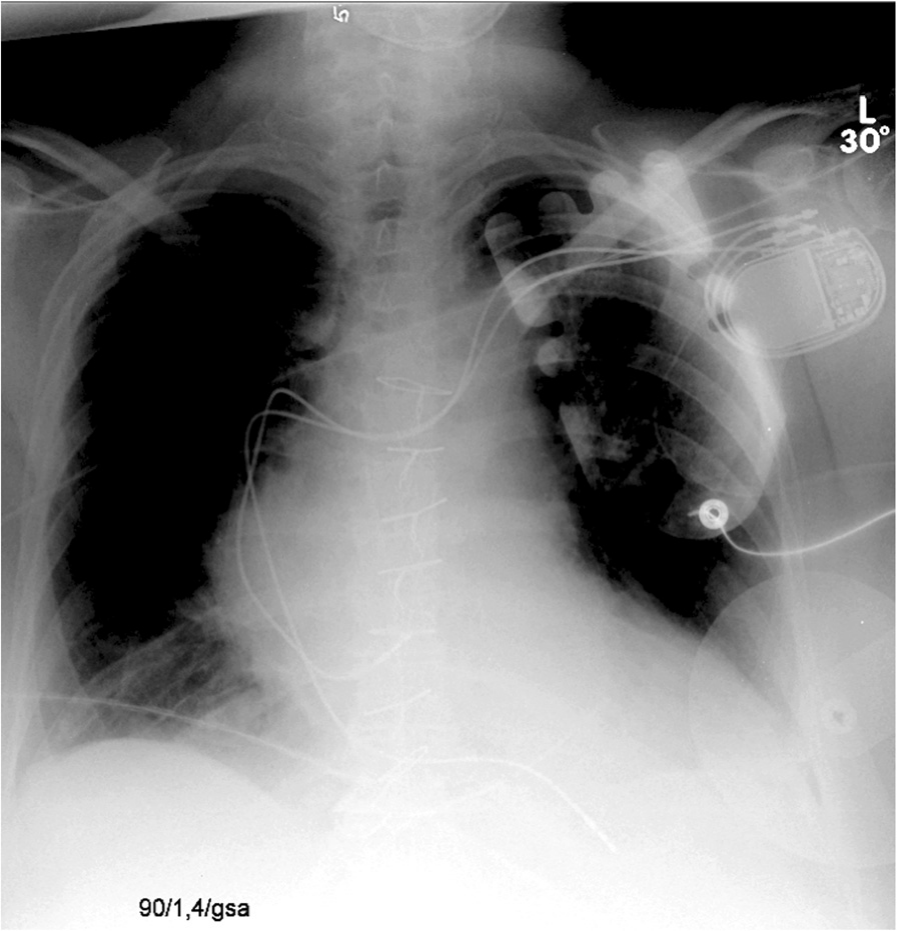
**Chest X-ray.** Atypical position of the atrial lead.

**Figure 2:**
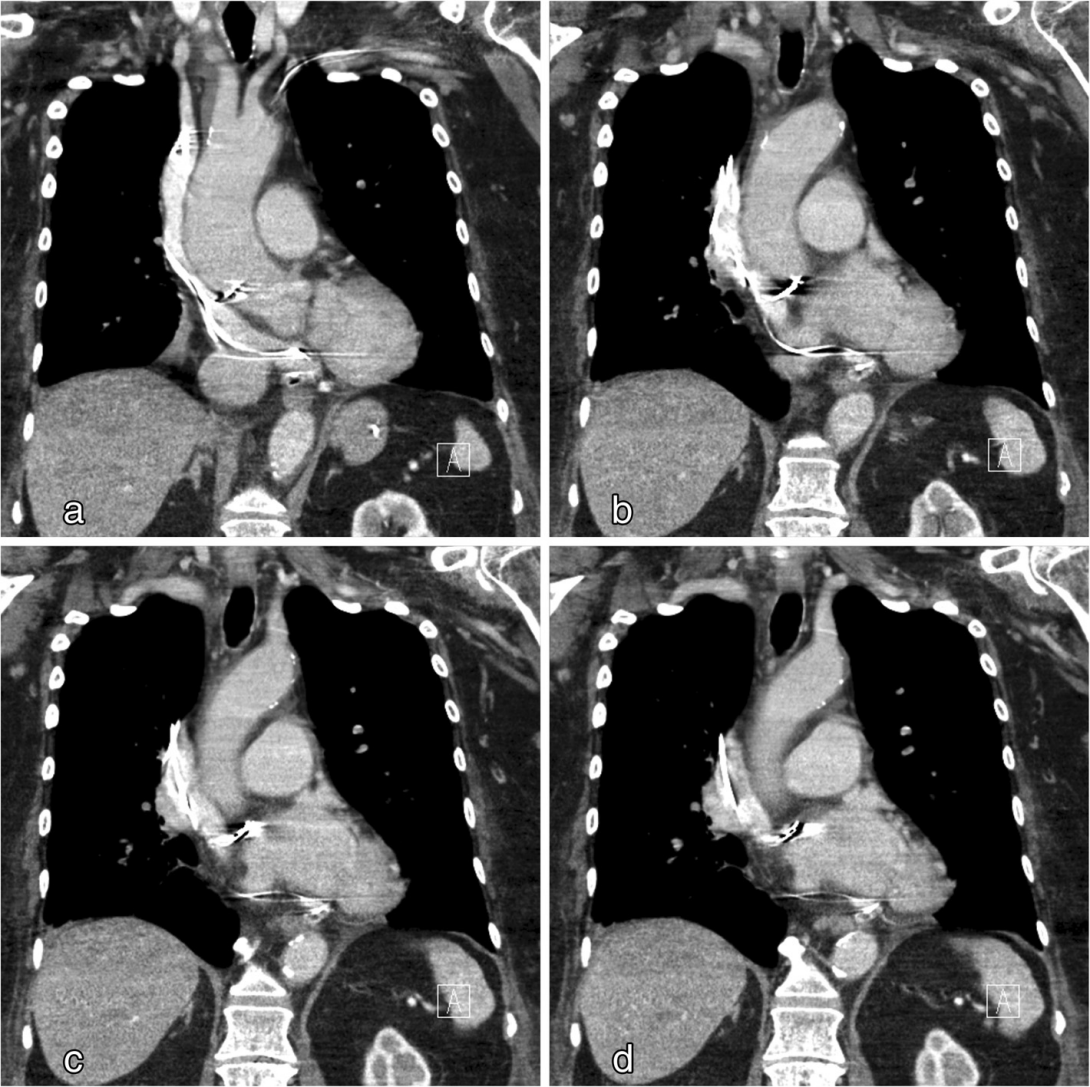
**Computed tomography scan.** The atrial lead connects to the roof of the left atrium; 2**a**-2**d** Four coronal CT slices, from ventral to dorsal.

On the second day after emergency surgery, an atypical position of the atrial lead was detected in routine chest X-ray (Figure 1). The subsequent chest CT scan revealed malpositioning of the atrial pacemaker lead. It was exiting the superior vena cava through a perforation, running in the transverse sinus dorsal of the ascending aorta and connecting to the roof of the left atrium (Figure 2).

**Figure 3:**
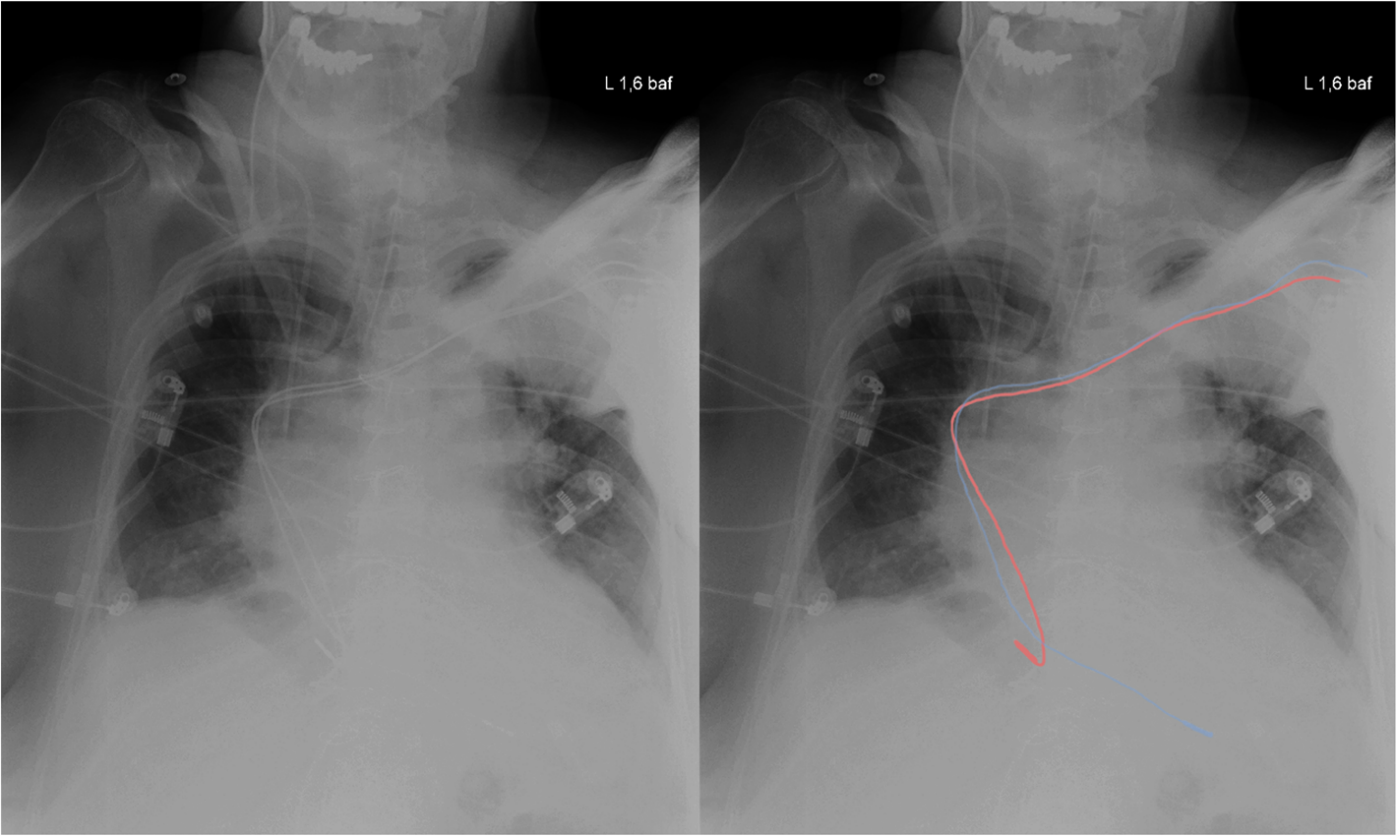
**Post-operative chest X-ray.** Both leads were re-positioned.

Based on these new findings, the patient underwent re-operation. Under general anaesthesia, in full preparation for re-sternotomy, an approach through the existing pacemaker wound was chosen. The atrial lead was withdrawn into the superior vena cava and re-positioned into the right atrial appendage. Again, the values for the lead were excellent (Table 1). Due to the slightly steep insertion into the apex, the ventricle lead was also re-positioned (Figure 3).

Over the entire course of the surgery and during follow-up, the fluid quantity in the drain was controlled to exclude any bleeding; a possible pericardial effusion was ruled out by echocardiography. The patient was transferred to the intensive care unit in stable haemodynamic conditions.

After an uneventful postoperative period the patient was discharged in excellent general condition.

## Conclusion

Perforation of a cardiac structure and subsequent tamponade is among the most serious adverse events of pacemaker implantation. Although it is a very uncommon complication, it may happen to any surgeon, independent of experience and skill level [4].

Perforations of cardiac structures and pericardial effusion are rare complications. In the FOLLOWPACE study, perforation of cardiac structures occurred in 0.40% and pericardial effusion in 0.13% of 1517 patients within 2 months after pacemaker implantation [2].

Our case was particularly deceptive as the values of the atrial lead showed inconspicuous parameters. The sensing through the lead was excellent with a P-value >3 mV and the stimulation threshold was <1 V at the time of implantation (Table 1).

Chest X-ray gave a first hint for malposition. The tip of the atrial lead usually curves upward for fixation in the atrial appendage, forming a “J”, while the ideal position for the right ventricular lead is located in the ventricular apex. As Figure 1 shows, the atrial lead in our patient is taking an atypical turn to the left.

Several risk factors for cardiac perforation have been identified in scientific literature. Steroid use within seven days prior to implantation (HR 3.2), temporary pacemaker wire placement (HR 2.7) and helical screw leads (HR 2.5) were increasing the likelihood for perforation in multivariate analysis [5]. Advanced age was also correlated to an increased risk for early pacemaker complications. For elderly patients (≥75 years of age) a significantly increased risk was found for any implantation complication compared to patients <75 years of age (5.1% vs. 3.4%, p = 0.006) [6].

The risk profile of our patient included increased age and the usage of helical screw leads.

Great vessel perforation is a complication of central venous access with an incidence of less than 1%. The vast majority of those perforations occurred when a right subclavian vein approach was chosen and could be linked to kinking of the guidewire during advancement of the vessel dilator [7].

To our best knowledge, only one case of superior vena caval perforation due to pacemaker placement was described in literature. *Fann et al.* reported problems with placing the lead, which was due to guidewire kinking [8].

We were not able to uncover the exact mechanism of perforation in our patient, but the initial surgeon reported the necessity of several approaches for lead positioning.

Meticulous attention should be paid to careful guidewire advancement through the subclavian vein. If unexpected resistance during guidewire placement occurs, the following manoeuvres may prevent major adverse events: Re-punctation, infusion of contrast dye to uncover vessel stenosis or occlusion, switch to the contralateral side and application of a long safe-sheath.

Perforation cannot be avoided completely, but the risk can be minimized with proper pre-operative assessment, intra-operative vigilance and post-operative control.

If the surgeon suspects malpositioning of a lead, immediate reaction is obligatory.

Although chest-X-ray for control of lead placement after pacemaker implantation is discussed controversially [9,10], it is a safe, cheap and readily available examination and may gives the hint for lead malposition. Utilization of elaborate imaging modalities, as echocardiography and thoracic CT scan, may be useful for subsequent definite diagnosis and for answering the crucial question, whether fast surgical revision is necessary.

Ideally, surgical backup and cardiopulmonary bypass standby are available at implanting centres to manage any bleeding at an early stage. Perforation and bleeding may not completely be avoidable, but subsequent complications like pericardial tamponade and cardiogenic shock are.

## Consent

Written informed consent was obtained from the patient for publication of this Case report and any accompanying images. A copy of the written consent is available for review by the Editor-in-Chief of this journal.

## Abbreviations

CPR: Cardiopulmonary resuscitation; CT: Computed tomography; HR: Hazard ratio.

## Competing interests

The authors declare that they have no competing interests.

## Authors’ contributions

CR: prepared the manuscript, drafted the manuscript. MA: participated in surgery, reviewed the manuscript. CK: operating surgeon, aided in literature search. DW: aided in literature search. AH: aided in literature search. AK: operating surgeon, reviewed the manuscript. All authors read and approved the final manuscript.

## Authors’ information

CR: PhD candidate, Department of Cardiac Surgery, Vienna General Hospital, Medical University of Vienna.

MA: resident, Department of Cardiac Surgery, Vienna General Hospital, Medical University of Vienna.

CK: Consultant, Department of Cardiac Surgery, Vienna General Hospital, Medical University of Vienna.

DW: Chief-resident, Department of Cardiac Surgery, Vienna General Hospital, Medical University of Vienna.

AH: PhD candidate, Department of Cardiac Surgery, Vienna General Hospital,Medical University of Vienna.

AK: Professor, Department of Cardiac Surgery, Vienna General Hospital, Medical University of Vienna.
